# Analysis of High Affinity Self-Association by Fluorescence Optical Sedimentation Velocity Analytical Ultracentrifugation of Labeled Proteins: Opportunities and Limitations

**DOI:** 10.1371/journal.pone.0083439

**Published:** 2013-12-17

**Authors:** Huaying Zhao, Suvendu Lomash, Carla Glasser, Mark L. Mayer, Peter Schuck

**Affiliations:** 1 Dynamics of Macromolecular Assembly Section, Laboratory of Cellular Imaging and Macromolecular Biophysics, National Institute of Biomedical Imaging and Bioengineering, National Institute of Health, Bethesda, Maryland, United States of America; 2 Laboratory of Cellular and Molecular Neurophysiology, Porter Neuroscience Research Center, National Institute of Child Health and Human Development; National Institute of Health, Bethesda, Maryland, United States of America; Johns Hopkins University, United States of America

## Abstract

Sedimentation velocity analytical ultracentrifugation (SV) is a powerful first-principle technique for the study of protein interactions, and allows a rigorous characterization of binding stoichiometry and affinities. A recently introduced commercial fluorescence optical detection system (FDS) permits analysis of high-affinity interactions by SV. However, for most proteins the attachment of an extrinsic fluorophore is an essential prerequisite for analysis by FDS-SV. Using the glutamate receptor GluA2 amino terminal domain as a model system for high-affinity homo-dimerization, we demonstrate how the experimental design and choice of fluorescent label can impact both the observed binding constants as well as the derived hydrodynamic parameter estimates for the monomer and dimer species. Specifically, FAM (5,6-carboxyfluorescein) was found to create different populations of artificially high-affinity and low-affinity dimers, as indicated by both FDS-SV and the kinetics of dimer dissociation studied using a bench-top fluorescence spectrometer and Förster Resonance Energy Transfer. By contrast, Dylight488 labeled GluA2, as well as GluA2 expressed as an EGFP fusion protein, yielded results consistent with estimates for unlabeled GluA2. Our study suggests considerations for the choice of labeling strategies, and highlights experimental designs that exploit specific opportunities of FDS-SV for improving the reliability of the binding isotherm analysis of interacting systems.

## Introduction

The cellular machinery of many biological functions is based on dynamic, reversible molecular interactions, and therefore characterization of the stoichiometry, specificity and cooperativity of protein-protein and protein-nucleic acid interactions is of key importance in cell biology. Such interactions include self-associations, two-component heterogeneous associations, as well as multi-protein assemblies. Sedimentation velocity (SV) analytical ultracentrifugation (AUC) has been applied to such studies in numerous biological systems, and provides exquisitely rich information on the properties of individual molecules, their complexes and their interactions [1]–[5]. A classic biophysical method which has seen substantial instrumental, theoretical, and computational improvements in recent years [6], SV has unique virtues for deciphering the binding properties of proteins in free solution, due to the strongly size-dependent movement of macromolecules in the centrifugal field, leading to hydrodynamic separation and characteristic patterns of co-migration that can be observed with high resolution. Two widely used conventional optical detection systems, an absorbance spectrophotometer and a Rayleigh interferometer, generally provide sufficient sensitivity for the label-free detection of macromolecules in the high nM to mM range. In favorable cases, concentrations as low as ∼10 nM (for a 50 kDa protein) can be reached with far UV absorbance [7], but the concomitant low signal/noise ratio limits data interpretation to analysis of signal weighted-average sedimentation coefficients. Therefore, for high affinity binding systems with *K_d_* in the low nM or even sub-nM regime, with conventional optical systems it is difficult to obtain an accurate sedimentation coefficient for the monomeric species, and for systems with a pM *K_d_* it may not be possible to see any dissociation of the oligomeric assemblies.

Fluorescent optical detection systems (FDS) for AUC that can overcome this limitation in sensitivity have been described [8], [9], and recently a commercial (Aviv Biomedical) FDS system with a fixed excitation wavelength of 488 nm was introduced [10], allowing measurements over the concentration range from ∼0.1 nM to ∼1 µM for suitably labeled molecules. Although FDS-SV was initially thought to be useful primarily for qualitative studies [9]–[11], there is increasing interest in using this approach for the quantitative analysis of macromolecular assemblies but few observations have been published. In a study of prototype applications for FDS-SV the detection of 1:1 but not 2:1 complexes for GFP binding to a high affinity anti-GFP monoclonal IgG antibody was reported [11], at conflict with the expected stoichiometry. In other reports FDS-SV seems to have worked well [12], [13]. However, a close examination of FDS-SV results from the self-association of the soluble amino terminal domain of the glutamate receptor GluA2 (GluA2 ATD) has highlighted methodological difficulties and yielded results inconsistent with those from other methods [7]. To establish methodology for the consistently reliable use of FDS-SV for the quantitative characterization of high-affinity self-and hetero-associating systems, we have recently described data analysis models for fluorescent-detected sedimentation velocity data that fold the specific properties of FDS data into the modeling of the evolution of signal profiles [14]. This can account, for example, for radial signal magnification gradients and temporal gradients of signal intensity arising from laser power drifts or photo-bleaching, as well as finite radial signal convolution and shadows at the base of the solution column [14], which otherwise can cause unsatisfactory fits and bias. For the first time, this allowed the modeling of FDS-SV data rationally, with signal/noise ratios comparable to or even exceeding those for conventional detection systems.

In the present work we have reexamined the abnormal sedimentation profile of the GluA2 ATD detected by FDS-SV and identified an effect of the fluorescent label, which is typically a pre-requisite for studying protein interactions by FDS-SV. The potential pitfalls of extrinsic labeling are well-known in the application of fluorescence techniques, including, as an extreme recent example, the artificial binding of 6-carboxyfluorescein (FAM) and FAM-labeled DNA to the DNA repair enzyme O(6)-alkylguanine-DNA alkyltransferase [15]. In a pioneering study of glutamate receptor amino terminal domains by FDS-SV [16], the same label was used, for which a subsequent study revealed abnormalities in the measured sedimentation coefficients, the origin of which was unknown [7]. With the large variety of commercially available fluorescent labels, it can be a non-trivial task to select those with physical and chemical properties that do not impact the interactions of interest. But, on the other hand, the ability to study high-affinity protein self-associations with FDS-SV opens the possibility for important applications that are not accessible in other popular biophysical techniques for analysis of protein interactions, such as surface plasmon resonance [17] or isothermal titration calorimetry [18], both of which are designed for the study of heterogeneous associations between dissimilar proteins but which are difficult to use to study homo-oligomerization. To examine specific problems and opportunities arising from the extrinsic labeling of proteins in FDS-SV, we used the nM-affinity homo-dimerization of GluA2 ATD as a model system, and conducted a comprehensive series of binding studies employing FAM at different labeling ratios; the structurally related label Dylight488; and the GluA2 ATD N-terminally fused to EGFP. When we compared the dimerization affinity, kinetics, and the best-fit sedimentation coefficients of monomer and dimer species between the different modified molecules, we observed a profound impact of FAM but not the other two labels. Drawing from this example, we present strategies for labeling and for the experimental design in the study of protein interactions by FDS-SV.

## Methods

### Protein Preparation

In brief, the GluA2 ATD including the native signal peptide (SP) was cloned into the pRK5-IRES-EGFP mammalian expression vector, with a C-terminal thrombin cleavage site and affinity tag (LVPRGS-His_8_) following the last native residue (Ser380). The protein was expressed in HEK293T cells by transient transfection of suspension cultures, and the secreted construct with native complex glycosylation was purified by affinity chromatography followed by ion exchange chromatography as described previously [7], [19]. The EGFP-GluA2 fusion construct (SP-linker-EGFP-linker-ATD-LVPRGS-His_8_) was created by overlap PCR using the EGFP A206K mutant to prevent dimer formation by GFP, and included an SGS tripeptide linker between the GluA2 native signal peptide and EGFP, and an SGSGS pentapeptide linker between EGFP and the Glu2 ATD.

### Protein Labeling

For labeling reactions, a 40 µM concentration of purified GluA2 ATD was mixed with amine reactive dyes, either *N*-hydroxysuccinimide (NHS) ester–activated Dylight488 (Thermo Fisher Scientific) or NHS Alexa Fluor 568 (Invitrogen) dissolved in DMSO and then resuspended in labeling buffer (20 mM Na_2_HPO_4_/NaH_2_PO_4_ pH 7.0, 150 mM NaCl, 1 mM EDTA); final label and DMSO concentrations were 100 µM and 250 µM, 2% and 4.9% DMSO (volume fraction), respectively. FAM (5,6-carboxyfluorescein, Biotium Inc) labeling was performed by mixing 10 µM protein with 130 µM NHS ester–activated dye (final DMSO concentration 0.3%). The reactions were incubated in the dark at room temperature; the incubation time was typically 1 h, but was varied between 0.5 – 2 h for study of the labeling ratio dependence of FAM. The reaction solution was then loaded onto a high resolution size exclusion chromatography column (Superdex 75 10/300 GL) equilibrated with labeling buffer at pH 7.5 to separate free dye from labeled protein. The protein concentration and labeling ratio were then determined by UV-Vis spectrophotometry using values for ε_280_ of 55,720 M^−1^cm^−1^ for the unmodified protein, ε_280_ of 10,290 M^−1^cm^−1^ and ε_493_ of 70,000 M^−1^cm^−1^ for Dylight488, ε_280_ of 15,910 M^−1^cm^−1^ and ε_495_ of 74,000 M^−1^cm^−1^ for FAM, ε_280_ of 41998 M^−1^cm^−1^ and ε_578_ of 91,300 M^−1^cm^−1^ for Alexa Fluor 568, ε_280_ of 73,605 M^−1^cm^−1^ and ε_488_ of 56,000 M^−1^cm^−1^ for EGFP. For the EGFP-GluA2 ATD, the labeling ratio is 1.0 since the fusion protein contains one molecule of EGFP A206K per construct.

### Fluorescence-Detected Analytical Ultracentrifugation (FDS-AUC)

Analytical ultracentrifugation (AUC) experiments were conducted in an Optima XL-A analytical ultracentrifuge (Beckman Coulter, Indianapolis, IN) equipped with a fluorescence detection system (AVIV Biomedical, Lakewood, NJ). Samples were prepared by dilution of concentrated protein stocks with labeling buffer at pH 7.5, with target concentrations for dilution series typically ranging from 0.1 nM to 2 µM labeled protein in the presence of 0.1 mg/mL of bovine serum albumin (BSA). For each titration series, the samples were prepared as a mixture of a constant low concentration (e.g. 0.5 nM) of the labeled protein with unlabeled protein at a wide range of concentrations. The protein samples were loaded into standard double-sector charcoal-filled epon centerpieces with 12-mm pathlength and either quartz or sapphire windows [1], [20]. Since no reference solution is required for FDS, both sectors were used for protein samples, with up to 14 samples per run using an 8-hole rotor.

Radial calibration was performed prior to each run at 3,000 rpm using the calibration cell filled with 10 µM fluorescein in Tris buffer (Tris 10 mM, KCl 100 mM, pH 7.80). At the same speed, PMT voltage and gain were adjusted for each cell, and focus scans were conducted for the samples with the lowest and highest concentration of labeled protein. An appropriate focusing depth was selected to maximize the signal and minimize inner filter effect for the high concentration sample, usually around 4,000 µm. The PMT setting was 34% or 38% in the current study. After these initial procedures, the centrifuge was stopped, samples were remixed by gently reversing the cells, and the cells were reloaded back into the rotor. The rotor was then temperature equilibrated to 20°C while resting in the centrifuge chamber for at least 3 hours after the console temperature reading showed 20.0°C. After acceleration to 50,000 rpm, the angles of data acquisition were verified to be centered and well within each sector. Data were acquired in 0.002 cm radial intervals with gain settings of 1 or 8 dependent on the signal.

In order to quantify signal contributions from the buffer components and BSA, a cell filled with the working buffer and 0.1 mg/mL BSA, respectively, in its two sectors was run along with other protein samples. We also performed control SV experiments for high concentration samples in theµM range for both labeled and unlabeled protein molecules using an AUC equipped with conventional Rayleigh interference and absorbance optics following a standard protocol [1].

### Ultracentrifugal Data Analysis

For the FDS-SV data, the sedimentation profiles were loaded to SEDFIT and automatically sorted at different gains while creating list-files for each sector and gain at given time span and intervals [14]. The sorted data were initially analyzed with the standard *c*(*s*) model [21] in SEDFIT version 14.3 (https://sedfitsedphat.nibib.nih.gov/software/default.aspx). For some of the data sets, the FDS specific computation tools in SEDFIT were engaged as described recently [14] to account for imperfections in alignment of the fluorescence optics, for temporal drifts of the signal, and for the shadow effect of the excitation beam close to the cell bottom. For these data sets, the quality of the fits was significantly improved after using the FDS tools. For data sets with low signal/noise ratio, the general *c*(*s*) analysis resulted in a sufficiently good fit quality, and thus no FDS tools were utilized. Meniscus positions and frictional ratios were treated as adjustable parameters in the non-linear regression of *c*(*s*).

The resulting *c*(*s*) distributions were subject to an analysis of the isotherm of signal weighted-average sedimentation coefficient (*s_w_*). The software GUSSI (http://biophysics.swmed.edu/MBR/software.html, kindly provided by Dr. Chad Brautigam) was used to plot and integrate the *c*(*s*) distributions between 2 S and 7 S to determine *s_w_* as a function of protein concentration. For the lowest concentrations where signals of the BSA carrier protein were detected in the control, corrections were applied to the *s_w_*-value to eliminate the carrier contributions with *s_w,adj_* =  (*s_w_×f_tot_* – *s_w,BSA_×f_tot,BSA_*)/(*f_tot_* – *f_tot,BSA_*) (where *f_tot_* and *f*_tot,BSA_ denote the total signal by integration of *c*(*s*) of the sample or BSA only, and *s_w_* and *s_w,BSA_* the corresponding weighted average sedimentation coefficients. The *s_w_* isotherm was loaded into SEDPHAT [22] for fitting with the homo-dimerization model:
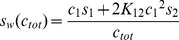
(1)


where *c_1_* and *c_tot_* indicate the molar concentration for monomer and total protomer, respectively, *K_12_* is the equilibrium association constant (*K_12_* = 1/*K_D_*), and *s_1_* and *s_2_* represent the *s*-values for monomer and dimer, respectively, under experimental conditions. *K_D_*, *s_1_* and *s_2_* were refined in the least-squares fit (and *c_1_* was implicitly determined through mass action law, given the known total concentration). For the analysis of multiple, chemically different populations of molecules, each representing an unknown fraction *f_i_* of the total material and each undergoing a monomer-dimer self-association with different affinity *K_12_^(i)^*, Eq. 1 was generalized to 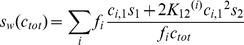
(2)with the constraints 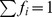 and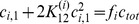, implemented in MATLAB (Mathworks, Natick, MA). All the experimental FDS-SV and SV data are plotted in units of experimental conditions. The error intervals of the best-fit *s*-values and *K_D_*-values were determined by error surface projection analysis [23] at a 95% confidence level.

### Kinetic Förster Resonance Energy Transfer (FRET) Experiments

FRET experiments were carried out at 20°C with excitation at 495 nm and emission recorded at 603 nm using a FluroMax 3 spectrofluorometer (Jobin/Yvon Horiba, Edison NJ). Proteins were labeled using hydroxysuccinimide ester–activated fluorophores, as described above. We studied three FRET donors: FAM-GluA2 ATD, Dylight488-GluA2 ATD and EGFP-GluA2 ATD, with Alexa Fluor 568 labeled GluA2 ATD used as the FRET acceptor. To establish the photo-stability of fluorophore labeled protein, the 603 nm emission was recorded for 4,000 sec from mixtures of 1 µM unlabeled protein to which labeled proteins at a total concentration of 100 nM were added prior to data acquisition. Following equilibration, this revealed a slow signal decrease with a best-fit rate of ∼1.5×10^−5 ^sec^−1^ when using FAM, and a much smaller signal decrease for Dylight488. For kinetic dissociation experiments, mixtures of acceptor and donor-labeled GluA2 ATD at 50 nM each were pre-equilibrated for 30 min (DL488) or 60 min (FAM), and then quenched by addition of 1 µM unlabeled protein while recording the resulting decrease of the 603 nm emission. The resulting kinetic data were corrected by subtraction of the baseline signal decrease recorded at the end of the experiment, and fit with exponential functions.

## Results

**Figure 1:**
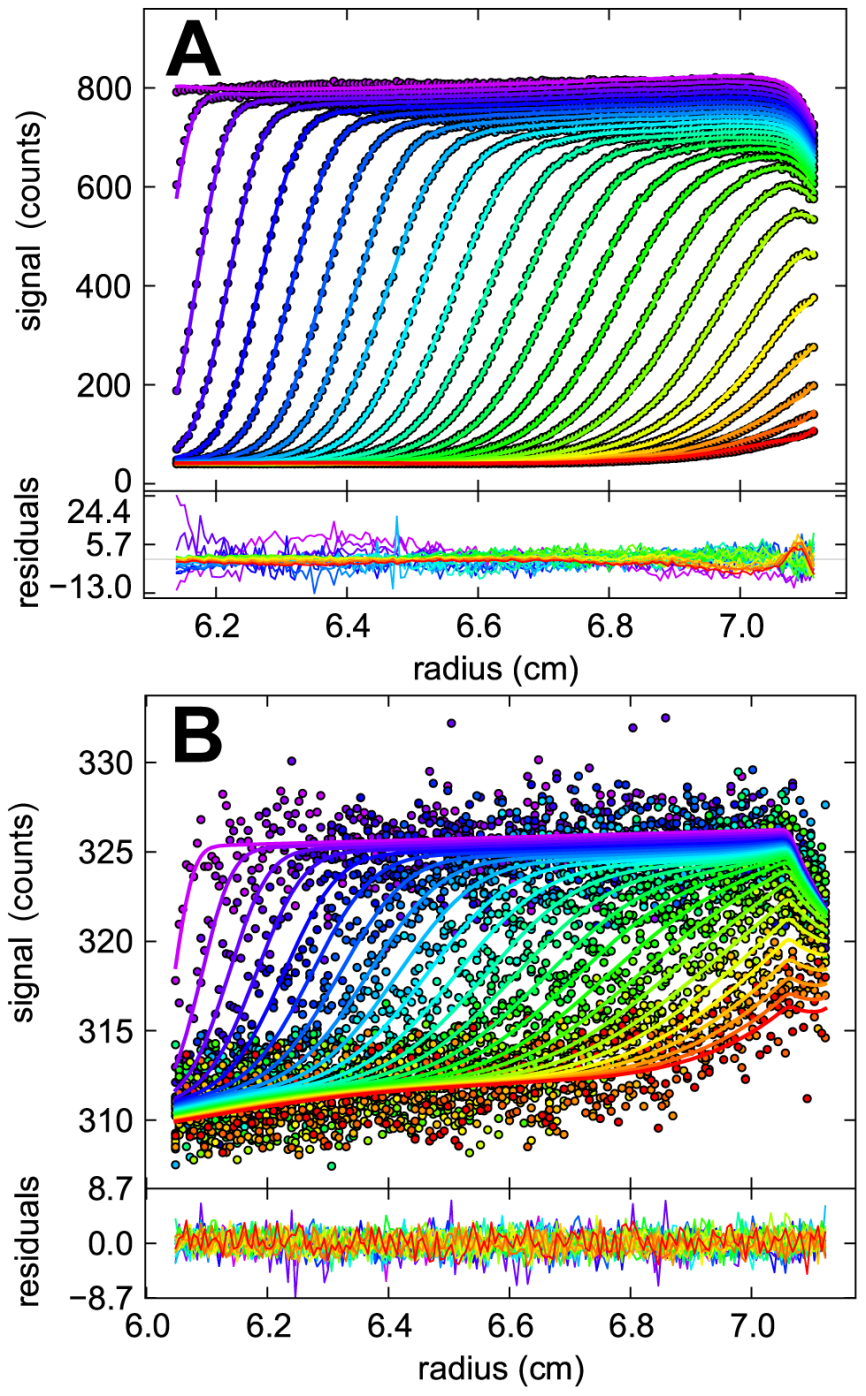
Examples of the evolution of radial signal profiles of GluA2 ATD. Circles are experimental data, for clarity showing only every 2nd data point of every 2^nd^ scan, and solid lines are the best-fit *c*(*s*) models, with increasing color temperature indicating later times. Residuals of the fits are shown in the small panels below the boundary profiles. (A) 300 nM of EGFP-GluA2 ATD, with a root-mean-square deviation (rmsd) of 2.6 signal units and a total signal of 755 units; (B) 1 nM Dylight488-GluA2 ATD with 9 nM unlabeled GluA2 ATD, with an rmsd of 1.566 signal units and a total signal of 13.9 units.

Similar to analysis by SV using interference or absorbance optics, the experimental FDS signal profiles of all samples were subjected to direct boundary analysis with distributions of Lamm equation solutions for non-interacting species, *c*(*s*). This is appropriate for the analysis of interacting components, because rapidly interacting systems represent effective particles [24] that to a sufficient approximation diffuse like non-interacting species [25], leading to a good fit of the sedimentation boundaries. As shown previously [22], due to the intimate relationship between integration of *c*(*s*)*ds* and the mass balance in the boundaries *c*(*r*)*rdr*, for the determination of rigorous *s_w_*-values, only a good representation of the experimental data by the boundary model is required, independent of chemical equilibria or reaction kinetics of the sedimenting species. Fits of excellent quality were consistently achieved over the entire concentration range for both dilution and titration series. For example, **Figure 1** shows the evolution of radial signal profiles and best-fit models for EGFP-GluA2 ATD at a relatively high concentration of 300 nM (Panel A), and for Dylight488-GluA2 ATD at a low concentration of 1 nM in the presence of 9 nM unlabeled protein (Panel B).

**Figure 2:**
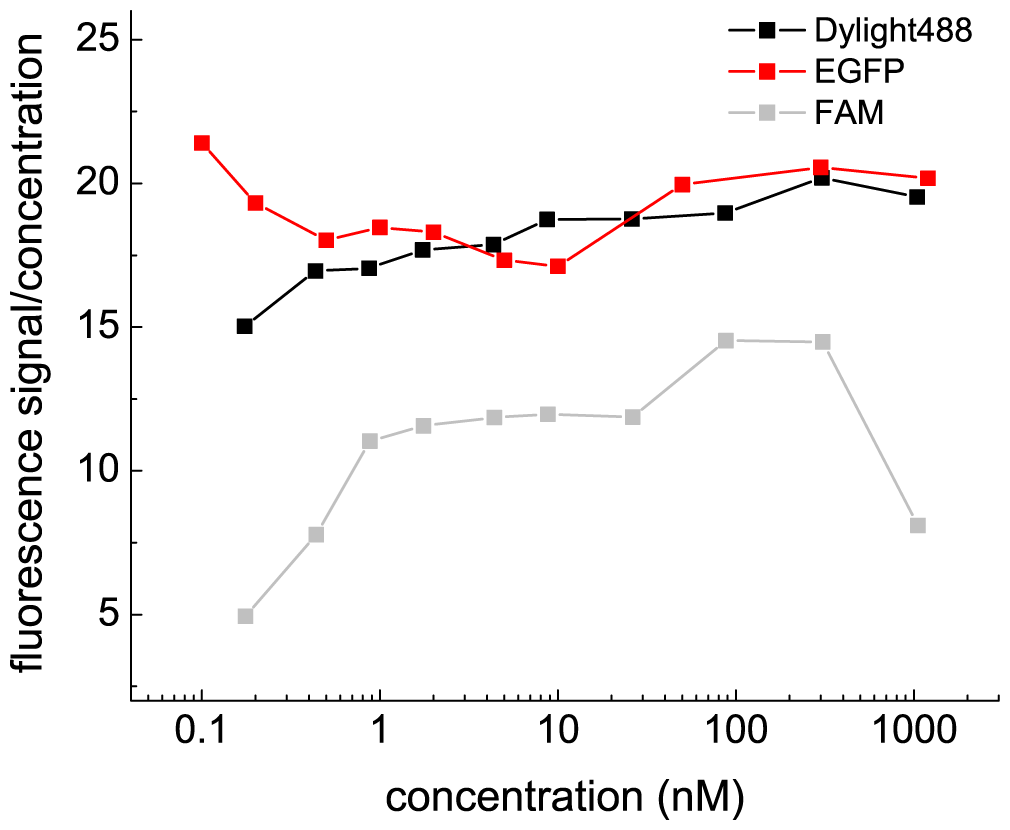
Total sedimentation boundary signals as determined from integration of the *c*(*s*) distribution, divided by the loaded protein concentration for GluA2 ATD labeled with Dylight488 at labeling ratio 1.43 (black), FAM at labeling ratio 1.32 (grey), and expressed as an EGFP fusion protein (red). Data were acquired at a constant PMT voltage of 36% (Dylight488 and FAM), or 38% (EGFP fusion protein) and signals were divided by the gain factor.

The dependence of the total measured boundary signal, at constant photomultiplier voltage and corrected for amplifier gain, on the concentration of loaded protein is shown in **Figure 2** for each of the three labels used for FDS-SV. When normalized relative to the loading concentration, this representation provides a sensitive control for the linearity of detection, for the loss of material due to the presence of rapidly sedimenting aggregates, and for changes in the quantum yield of the fluorophor with association state. None of these factors appears to be significant, considering imperfections from dilution errors over 4 orders of magnitude. A small decrease in the molar signal increment at the lowest concentration for FAM-labeled GluA2 might indicate imperfect blocking of surface adsorption sites by BSA, but this would produce only a slight underestimate of the *K_D_*, by less than a factor of two. No such change of the specific signal increment paralleling the binding isotherm was observed for Dylight488 or EGFP-labeled GluA2 ATD.

**Figure 3:**
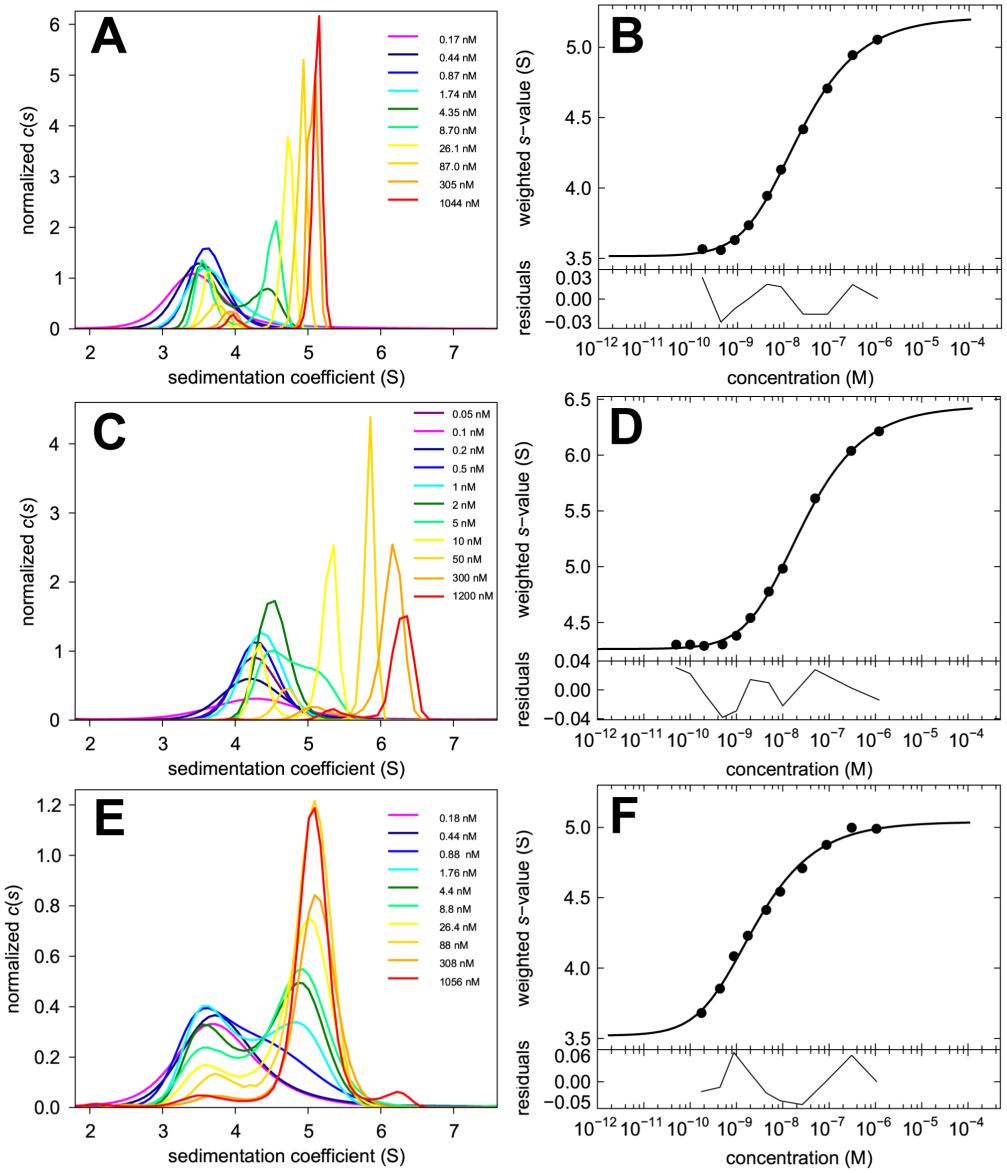
Dilution series with different fluorescent labels for the GluA2 ATD. Sedimentation coefficient distributions *c*(*s*) (Panels A, C, and E) and the resulting *s_w_* isotherms (Panels B, D, and F) from integration of *c*(*s*) for Dylight488-GluA2 ATD (first row), EGFP-GluA2 ATD (second row) and FAM-GluA2 ATD (third row). In the *c*(*s*) plots, the distributions were normalized with respect to the loading concentrations indicated. In the isotherm plots, solid circles are the *s_w_* data from the dilution series, and the solid line is the best-fit isotherm with a monomer-dimer model, resulting in best-fit estimates of *K_D_* 20.5 nM (95% CI 15.9 – 26.4), *s*_1_ = 3.52 S (95% CI 3.47 – 3.56), and *s*_2_ = 5.21 S (95% CI 5.15 – 5.28) for Dylight488-GluA2 ATD; *K_D_* = 25.4 nM (95% CI 20.1 – 31.9), *s*_1_ = 4.26 S (95% CI 4.22 – 4.30), and *s*_2_ = 6.44 S (95% CI 6.35 – 6.53) for EGFP-GluA2 ATD; and *K_D_* = 2.3 nM (95% CI 0.99 – 5.0), *s*_1_ = 3.52 S (95% CI 3.22 – 3.72), and *s*_2_ = 5.04 S (95% CI 4.95 – 5.14) for FAM-GluA2 ATD.

The family of *c*(*s*) distributions and the isotherm of *s_w_* as a function of concentration for the dilution series of Dylight488-GluA2 ATD is shown in **Figure 3A and B**, respectively. From the characteristic concentration-dependence of the peak position of *c*(*s*), especially at higher concentrations where the signal/noise ratio would be sufficient to resolve co-existing non-interacting monomer and dimer species, we can conclude that the dissociation relaxation time constant is small compared to the time-scale of the sedimentation experiment [26], consistent with a lifetime of 170 sec determined subsequently by FRET experiments (see below). It should be noted that the low-*s* peak at ∼3.5 – 4 S of the normalized *c*(*s*) distributions at concentrations substantially above the *K_D_* does not reflect impurities or non-reactive monomer, but a fraction of dimeric protein that invariably dissociates during the sedimentation process due to the lower concentration in the trailing edge of the diffusion-broadened sedimentation boundary, as predicted by theory and seen in simulations of rapid monomer-dimer systems [22]. These features are revealed by the excellent signal/noise ratio for data in the dilution series, but are not visible in the *c*(*s*) distributions from the corresponding titration series of a constant low concentration of Dylight488-GluA2 ATD with unlabeled GluA2 ATD (**Figure S1A**), due to the broadening effect of regularization at the constant low signal/noise ratio that obscures any characteristic kinetic effect in the sedimentation pattern.

Analysis of the *s_w_* binding isotherm from the dilution series data of Dylight488-GluA2 ATD (solid symbols and line in **Figure 3B**) leads to a best-fit estimate for the dimerization equilibrium constant *K_D_* of 20.5 nM (95% CI 15.9 – 26.4). This is approximately a factor two higher, but within error consistent with the previously determined *K_D_* estimate of 9.4 nM for unlabeled GluA2 ATD determined using conventional far-UV absorbance optics [7]. However, in contrast to the absorbance data for which the monomer *s*-value was constrained using values derived from hydrodynamic modeling, from the close to four orders of magnitude span of concentrations studied for the FDS data, both *s*_1_ = 3.52 S (95% CI 3.47 – 3.56) and *s*_2_ = 5.21 S (95% CI 5.15 – 5.28) are very well-determined, leading to a dimer-to-monomer ratio of *s*-values of 1.48. Likewise, the weighted-average monomer and dimer *s*-values of 3.48 S (95% CI 3.36 – 3.58) and 5.14 S (95% CI 5.08 – 5.21) obtained from the titration experiment (**Figure S1B**), which gave a *K_D_*-value of 16.5 nM (95% CI 10.9 – 24.4), are also well determined and highly consistent with the dilution data.

Analogous results were obtained in the dilution experiment with EGFP-GluA2 ATD (**Figure 3C and 3D**). The best-fit isotherm of the dilution data led to a *K_D_* estimate of 25.4 nM (95% CI 20.1 – 31.9), which is consistent with the Dylight488-GluA2 ATD result, but due to the significantly increased molecular mass of the EGFP fusion protein, not surprisingly, the *s*-values were substantially higher. Again, the fitted monomer and dimer *s*-values were very well defined with *s*_1_ = 4.26 S (95% CI 4.22 – 4.30), and *s*_2_ = 6.44 S (95% CI 6.35 – 6.53). Also consistent with the results from Dylight488-GluA2 ATD data, the corresponding titration series (**Figures S1C and S1D**) yielded similar results with a best-fit *K_D_* of 22.0 nM (95% CI 16.3 – 29.6). Finally, similar to the Dylight488-labeled protein, the dimer-to-monomer ratio of *s*-values for the EGFP fusion protein is 1.51 compared to the value of 1.48 for the dye-labeled protein.

FAM, the third label used in the present work, was used in the original analysis of glutamate receptor ATD oligomerization [7], [16]. A dilution isotherm experiment with FAM-GluA2 ATD is shown in **Figure 3E/F**. Overall, from the relative independence of *c*(*s*) peak positions on protein concentration shown **Figure 3E**, we can conclude that the life-time of the dimer is significantly longer that of Dylight488-GluA2 ATD or EGFP-GluA2 ATD. This feature is consistent with the *c*(*s*) distributions reported previously for FAM-GluA2 ATD by Rossmann et al. [16] and reproduced by us in subsequent experiments [7]. Also, it is notable that even at concentrations as low as 0.2 nM and 0.4 nM the *c*(*s*) distributions are broad, with asymmetric peaks, suggesting the presence of multiple species, in contrast to the expectation that the protein should be predominantly monomeric based on the *K_D_* determined above. Furthermore, for the highest concentration of 1,200 nM a small population of faster sedimenting species at 6.2 S can be observed, far above the highest *s*-value of the similar-sized Dylight488-GluA2 ATD. However, in different FAM-GluA2 ATD batches with different labeling ratios (see below) this trace species was not consistently observed.

The isotherm analysis of *s_w_*-values for FAM-GluA2 ATD shown in **Figure 3F** exhibits a mid-point at noticeably lower concentrations than in **Figure 3B** or **Figure 3D**, with a best-fit *K_D_* of 2.3 nM (95% CI 0.99 – 5.0), approximately 10-fold smaller than that determined for Dylight488-GluA2 ATD and EGFP-GluA2 ATD, but in good agreement with the value of 1.8 nM reported previously for FAM-labeled GluA2 ATD [16]. However, over the concentration range used in this experiment the end-points of the isotherm are not as well defined as for the DL-488 and EGFP labeled GluA2 ATD isotherms shown in **Figure 3B** and **Figure 3D**. The estimate from the isotherm analysis for the FAM-GluA2 ATD monomer *s*-value is 3.52 (95% CI 3.22 – 3.72) S, consistent with that of Dylight488-GluA2 ATD, and for the dimer *s*-value it is 5.04 (95% CI 4.95 – 5.14) S, lower than the value for Dylight488-GluA2 ATD. Together, the ratio of dimer *s*-value to monomer *s*-value is only 1.43, lower than that obtained for Dylight488-GluA2 ATD, and also lower than the value predicted by hydrodynamic modeling. The corresponding titration series for 1 nM FAM-GluA2 ATD with increasing concentrations of unlabeled protein is shown in **Figures S1E and S1F**. The best-fit *K_D_* value of the titration *s_w_* isotherm (**Figure S1F**) was 9.0 nM (95% CI 3.9 – 18.0), noticeably higher but statistically consistent within a 95% confidence interval with the dilution series.

**Figure 4:**
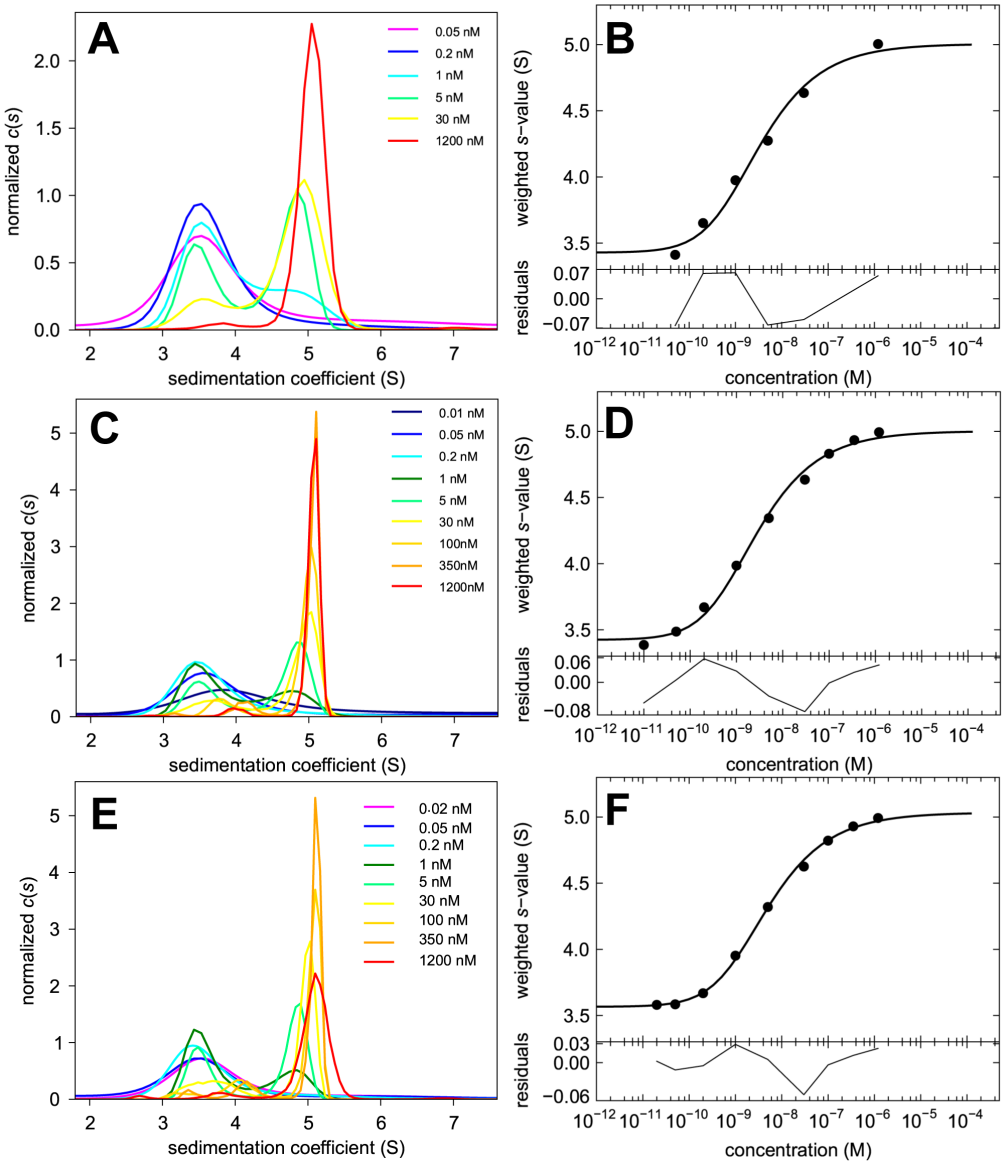
Dilution series for FAM-GluA2 ATD at different labeling ratios. Shown are the sedimentation coefficient distribution distributions *c*(*s*) (Panels A, C, and E) and the resulting *s_w_* isotherms (Panels B, D, and F) from integration of *c*(*s*) for FAM-GluA2 ATD at labeling ratios of 0.68 (first row), 1.05 (second row) and 2.26 (third row). Analogous to Figure 3, in the *c*(*s*) plots the distributions were normalized with respect to the loading concentrations indicated, and in the isotherm plots, solid circles are the *s_w_* data from the dilution series, and the solid line is the best-fit isotherm with a monomer-dimer model. This resulted in best-fit values at the labeling ratio of 0.68 (Panel B) of *K_D_* = 3.1 nM (95% CI 0.16 – 30.5), *s*_1_ = 3.43 S (95% CI 2.53 – 3.82), and *s*_2_ = 5.01 S (95% CI 4.60 – 5.51); for the labeling ratio of 1.05 (Panel D) the best-fit values were *K_D_* = 2.6 nM (95% CI 1.1 – 5.9), *s*_1_ = 3.42 S (95% CI 3.27 – 3.56), and *s*_2_ = 5.00 S (95% CI 4.88 – 5.14); and for the labeling ratio of 2.26 (Panel F) the best-fit values were *K_D_* = 4.7 nM (95% CI 3.0 – 7.3), *s*_1_ = 3.57 S (95% CI 3.49 – 3.63), and *s*_2_ = 5.03 S (95% CI 4.96 – 5.11).

The apparent inconsistency of the affinity and kinetics of dimerization of FAM-GluA2 ATD compared to the behavior of Dylight488-GluA2 ATD and EGFP-GluA2 ATD suggest an alteration of the binding properties of the protein due to the attached dye. To examine this further we carried out a study of FAM-GluA2 ATD with different label ratios. Through variation of the incubation time of the cross-linking reaction, labeling ratios of 0.68, 1.05, and 2.26 were achieved. For each preparation a binding experiment by FDS-SV was carried out using a dilution series, similar to that shown in **Figure 3E/F**, but in order to define better the monomer *s*-value we included extremely low concentrations, in some cases as low as 10 pM to 50 pM. The *c*(*s*) distributions and *s_w_* isotherms are shown in **Figure 4**. Again, no shift in *c*(*s*) peak position with concentration can be discerned at any of the labeling ratios, suggesting the presence of slowly exchanging monomeric and dimeric states with concentration-dependent populations. No significant difference in the *K_D_* estimate was found in any of these experiments relative to the FAM-GluA2 ATD data shown in **Figure 3**, with values of 3.1, 2.6 and 4.7 nM for labeling ratios of 0.68, 1.05 and 2.26 respectively.

Next, we focused on the potential role of BSA, which was used as a carrier protein to prevent surface adsorption of GluA2 ATD, and considering the well-known capacity of serum albumin to bind different small molecule ligands [27], asked whether it could be interacting with a ‘sticky’ label such as FAM. To this end, we conducted additional control experiments with different concentrations of BSA (0.1 – 1.0 mg/ml) in the presence of 1 nM and 100 nM FAM-GluA2 ATD at the highest labeling ratio. Neither the *c*(*s*) distribution (**Figure S2**), nor the *s_w_*-value revealed any significant dependence on the concentration of BSA (**Table S1**). However, a further control experiment with 100 nM FAM-GluA2 ATD in the absence of BSA did reveal a signal loss of ∼ 25% (data not shown). This confirms that the presence of BSA, or another carrier protein, that is inert to the sedimentation coefficient distribution of the labeled protein, is essential for conducting FDS-SV experiments without signal loss due to non-specific adsorption presumably to surfaces of the cell assembly [11]. However, when working with FDS detection conditions suitable for very low protein concentrations we observed small fluorescence signal contributions from BSA, for which the above *s*_w_ data were corrected as described in the Methods. In the present study, this amounted to very small corrections of –1 to –4% at 0.2 nM Dylight488-GluA2 ATD, but a larger correction of –8% at 0.02 nM FAM-GluA2 ATD.

**Figure 5:**
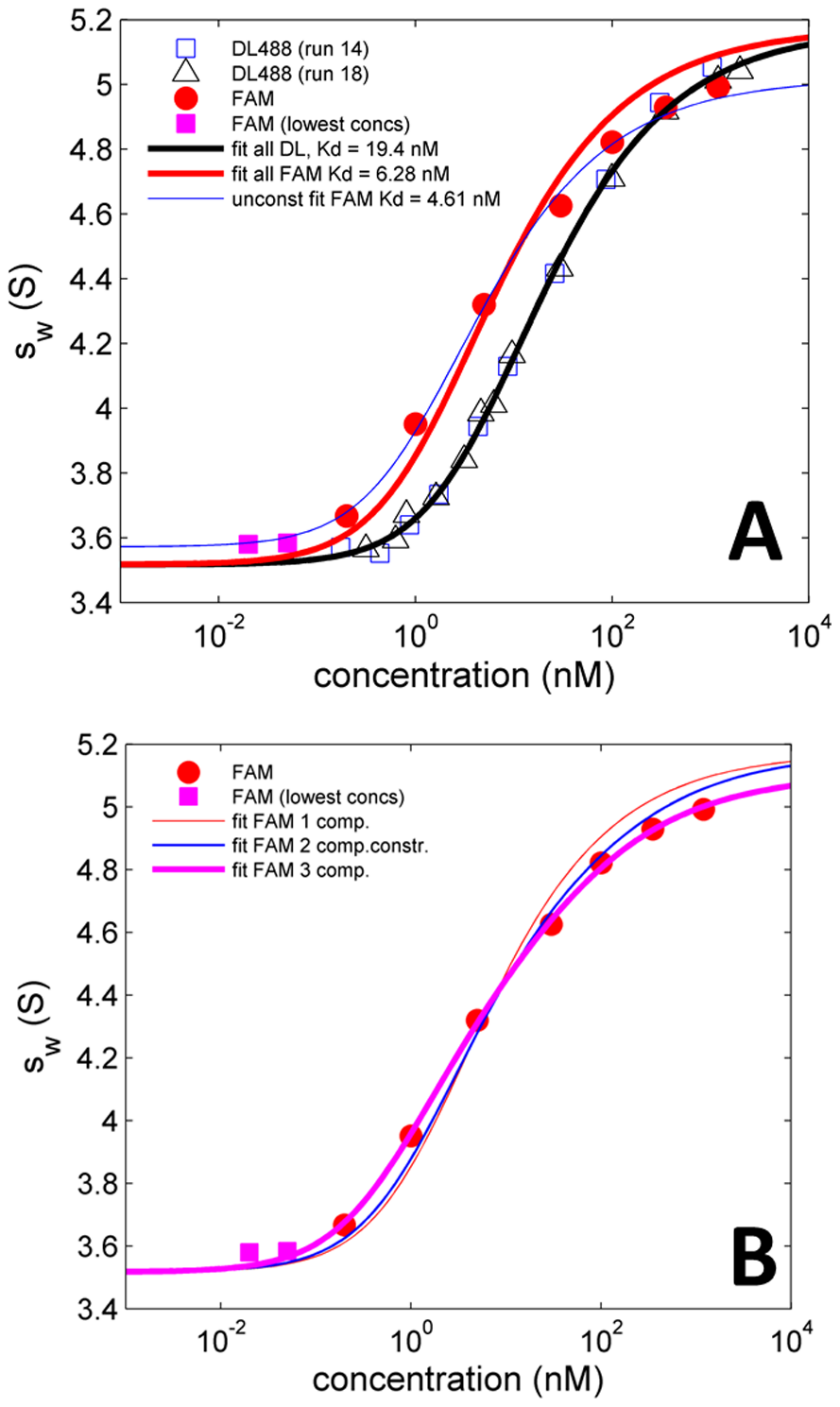
Comparison of the *s_w_* isotherm of Dylight488-GluA2 ATD with that of FAM-GluA2 ATD. (A) Dilution series of Dylight488-GluA2 ATD (open squares and triangles show data from two different runs) with a global best-fit single component, single class of sites model (bold black line) which leads to estimates of *K_D_* = 19.4 nM, *s*_1_ = 3.52 S, and *s*_2_ = 5.17 S, with a rmsd of 0.021 S. A dilution series of FAM-GluA2 ATD with a labeling ratio 1.32 is shown in solid red circles, extended to lower concentrations as shown with bold magenta squares. A single-component, single-site fit to the entire FAM-GluA2 ATD data, constrained to the *s*-values derived from the Dylight488-GluA2 ATD analysis, is shown as red solid line, leading to a best-fit *K*_D_–value of 6.3 nM and rmsd of 0.077 S. For comparison, a single-component fit to the FAM-GluA2 ATD data excluding the two lowest concentrations (i.e. a fit to the red circles only) and freely adjusting the *s*-values, is shown as thin blue line, resulting in a *K*_D_ estimate of 4.6 nM and apparent *s*_1_- and *s*_1_-values of 3.58 S and 5.02 S, respectively, with an rmsd of 0.023 S. (B) The same FAM data as in Panel A, modeled with different numbers of components, each exhibiting a different class of sites, and each constrained to have the same monomer and dimer *s*-value as derived from the single-component fit to Dylight488-GluA2 ATD shown in Panel A. (1) For comparison, the single component fit from Panel A (thin red line, rmsd  =  0.077S); (2) A two-component model (thin blue line) with *K*_D_ values constrained to be 1.7-fold above and 4.5-fold below that of Dylight488-GluA2 ATD, as may be suggested by the dissociation kinetics observed in the FRET experiment (thin blue line, rmsd  =  0.048 S); (3) A three component fit (bold magenta line) with one component exhibiting a higher affinity dimerization (best-fit *K_D_*  =  1.8 nM, comprising 75% of molecules), one component constrained to be equivalent to that of Dylight488-GluA2 ATD (21% of molecules), and one component with weaker dimerization (best-fit *K_D_*  =  24 µM, comprising 4.4% of molecules), leading to a best-fit rmsd of 0.018 S.

We next directly compared the isotherm of FAM-GluA2 ATD with that of Dylight488-GluA2 ATD (**Figure 5A**). It can be discerned that in addition to a leftward shift, the transition from monomer to dimer is broader and shallower for FAM-GluA2 ATD, with a single component fit leading to systematic deviations and a root-mean-square deviation (rmsd) of 0.077 S (red and magenta solid symbols, and red line) while for Dylight488-GluA2 ATD the single-component model fit is excellent with an rmsd of only 0.021 S (black and blue open symbols, and black line); this difference likely indicates the presence of multiple classes of molecules with different dimerization energies for the FAM labeled protein. (It should be noted that the different *K_D_* for FAM-GluA2 ATD in **Figure 5A** compared to **Figure 3** arisesfrom constraints in the model of **Figure 5** to the *s*-value estimates derived from Dylight488-GluA2 ATD.) As a plausible, simple model we hypothesized the presence of molecules that are identical in binding properties to the unlabeled protein (taken to be the same as that measured for Dylight488-GluA2 ATD), one class that is of higher binding energy (motivated by the requirement for the isotherm midpoint to be at lower concentrations than for Dylight488-GluA2 ATD), and one class that shows weaker dimerization (motivated by the lack of saturation and shallow increase at the highest concentrations). It was assumed that all of these populations are hydrodynamically identical to the respective monomer and dimer species of the native protein and have the same *s*-values for monomer and dimer as determined for Dylight488-GluA2 ATD. This resulted in an excellent fit (magenta line in **Figure 5B**, rmsd  =  0.018 S), with the high-affinity population representing 75% with *K_D_* = 1.8 nM, and the low-affinity population representing 4.4% with a best-fit but ill-defined *K_D_* of 24 µM, with the remaining species accounted for by a 'native' population of *K_D_* = 20.5 nM, the value determined for Dylight488 labeled GluA2 ATD. A slightly lower quality of fit, rmsd  =  0.022 S, was achieved considering only the high-affinity and ‘native’ interactions (data not shown).

**Figure 6:**
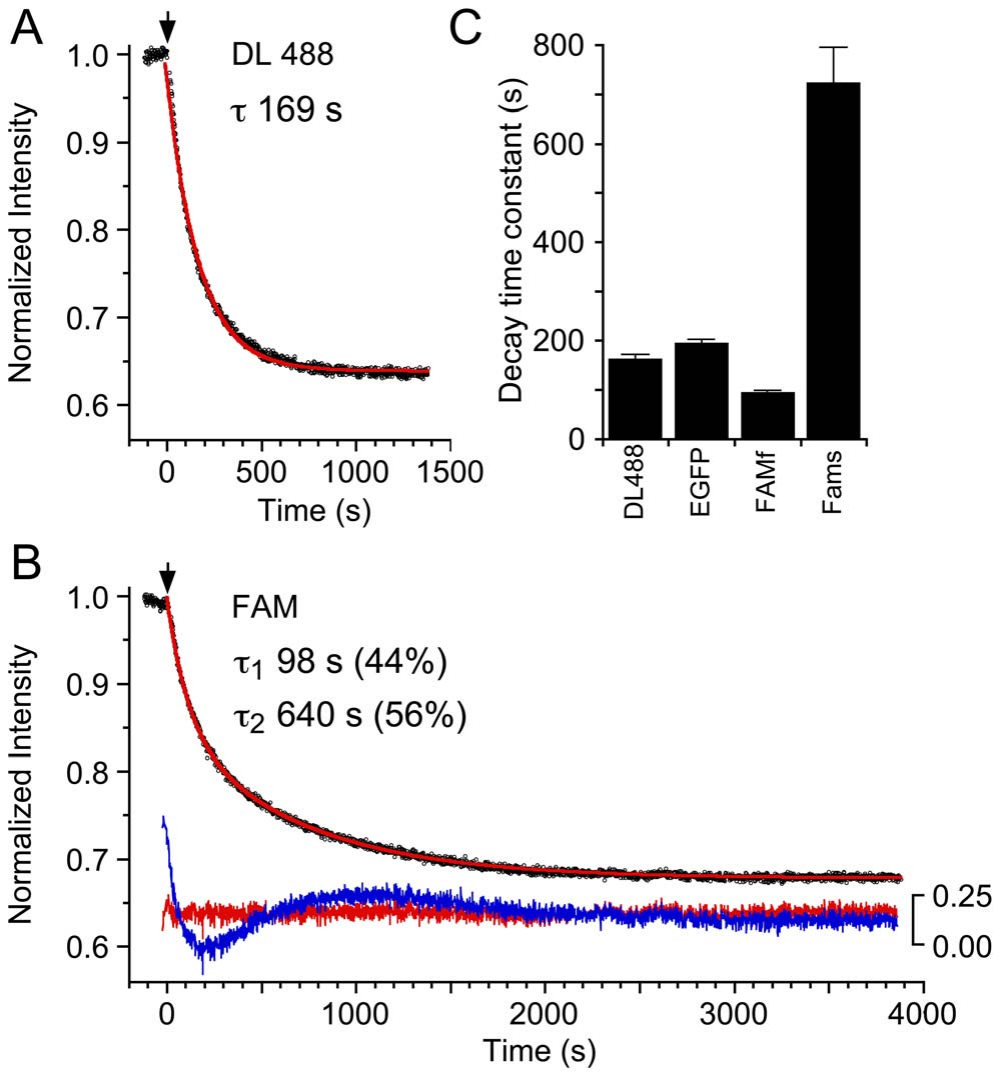
Analysis of GluA2 ATD dimer dissociation kinetics measured by FRET. (A) Decay of emission at 603 nm after addition of 1 µM unlabeled GluA2 (arrow) to a pre-equilibrated mix of 50 nM Dylight488-GluA2 ATD and 50 nM AF568-GluA2 ATD; the red line shows a single exponential fit of time constant 169 s, with decay to 64% of the initial amplitude. (B) Decay of emission at 603 nm after addition of 1 µM unlabeled GluA2 (arrow) to a pre-equilibrated mix of 50 nM FAM-GluA2 ATD and 50 nM AF568-GluA2 ATD; the red line shows a double exponential fit of time constant 98 s (44%) and 640 s (56%), with a total decay to 68% of the initial amplitude; the lower pair of red and blue lines show residuals on an expanded scale for double (χ^2^ 18.2×10^−3^) and single (χ^2^ 82.1×10^−3^) exponential fits, respectively. (C) Bar plot summarizing mean decay time constants for AF568-GluA2 FRET signal after addition of 1 µM unlabeled GluA2 ATD with either Dylight488-GluA2 ATD, EGFP-GluA2 ATD, or FAM-GluA2 ATD as the FRET donor; error bars show SD (n = 3).

To obtain independent evidence for FAM-GluA2 ATD species heterogeneity, and to directly estimate dimer lifetimes for the Dylight488-GluA2, EGFP-GluA2, and FAM-GluA2 ATDs, we carried out FRET experiments in a benchtop spectrofluorometer, using AF568-GluA2 ATD as an acceptor and either FAM, Dylight488 or EGFP labeled GluA2 ATD as donor. In experiments where 50 nM donor-labeled GluA2 ATD and 50 nM acceptor-labeled GluA2 ATD were pre-equilibrated to allow formation of hetero-dimers, the addition of 1 µM unlabeled GluA2 ATD produced a time-dependent decrease in the FRET signal. This is because the addition of excess unlabeled GluA2 leads to formation of unlabeled/labeled dimers as the majority species for labeled GluA2 ATDs. In this configuration, due to the high concentration of excess unlabeled GluA2 ATD, the corresponding decrease of the FRET signal reports mainly on the lifetime of donor/acceptor heterodimers. For the Dylight488-GluA2 and EGFP-GluA2 ATDs, the resulting data were well-described by a single-exponential process with a half life of 163 ± 9 and 196 ± 6 sec (mean ± SD, n = 3) respectively (**Figure 6AC**), consistent with the sedimentation boundary patterns reported above for Dylight488- and EGFP-GluA2 (**Figure 3A and 3C**).

By contrast, with FAM-GluA2 ATD as a donor, a multi-exponential decay was observed (**Figure 6B**). After subtraction of an extremely slow component likely representing photo-bleaching of FAM (slope ∼–1.5×10^−5^ sec^−1^), that was recorded also for pre-equilibrated mixtures of the same composition, the resulting data were well described by exponential fits with life-times of 95 ± 3 sec and 723 ± 72 sec (n = 3), approximately equally populated. If the effect of FAM labeling was only to affect the off-rate constant, then this would correspond to a combination of ∼1.7-fold weaker and ∼4.5-fold higher affinity than Dylight488-labeled GluA2 ATD. This is remarkably consistent with the conclusions drawn from the sedimentation experiments of FAM- and Dylight488-labeled GluA2 ATD. A fit of the FAM-GluA2 *s*_w_-isotherm with a model of two populations constrained to be 1.7-fold weaker and 4.5-fold higher affinity (**Figure 5B**, thin blue line), produces a significantly better model, rmsd 0.048 S, than a single population model (**Figure 5B**, thin red line), rmsd 0.077 S, but is significantly less good than the model with three populations of different affinity (**Figure 5B**, bold magenta line), which produces a fit with rmsd of only 0.018 S. This points to the possibility that dimers of two FAM-GluA2 ATD molecules may behave even more differently from unlabeled protein than dimers containing only a single FAM-GluA2 ATD molecule, as suggested also by the slight difference between dilution and titration isotherms of FAM-GluA2 ATD in SV.

## Discussion

The present work re-emphasizes the need for caution and control experiments when working with modified proteins in studies aimed at determining their interaction properties, and highlights experimental designs of FDS-SV studies that minimize artifacts and offer diagnostics for their detection. When studying simple two-component heterogeneous associations, it is frequently possible to consider the labeled protein as a third component, and to extract from experiments with mixtures of labeled and unlabeled proteins and their unlabeled binding partner information on the binding affinities between pairs of unlabeled proteins. Unfortunately, this is not possible for self-associations, mixed self- and hetero-associations, or multi-component interactions of three or more proteins. For self-associations one can still adopt the strategy of comparing isotherms for a dilution series of labeled protein with the results from a titration series of a constant concentration of labeled protein with a range of unlabeled protein concentrations. However, since in this case at least one binding partner of the monitored reaction is modified, only certain types of artifacts may be flagged. Furthermore, due to the intrinsically lower signal/noise ratio of data from a titration series, kinetic information and detailed information on trace components available in *c*(*s*) at higher signal strengths are obscured, and only weighted-average sedimentation coefficients can be extracted. In the present study of GluA2 ATD homo-dimerization, only small differences that were statistically insignificant at the 95% confidence level were measured when comparing dilution and titration isotherms for FAM-labeled protein, and thus this comparison was insufficient to reveal the effect of the FAM label.

At all labeling ratios tested, FAM-labeled preparations behaved as an ensemble of molecules with different binding affinity, leading to a significantly broader binding isotherm than would be expected for a single-component homo-dimerization process. This conclusion from SV was corroborated independently by measuring the dissociation kinetics of FAM-labeled GluA2 ATD by FRET. When the experimental SV data were constrained to the transition region, for example, following the binding process from 0.1-fold *K_D_* to 10-fold *K_D_* as is typically considered adequate for studying unlabeled proteins using conventional detection [1], a reasonable fit was achieved with a standard single component, single class of sites model (thin blue line, **Figure 5A**), but the resulting apparent affinity and *s*-values were dependent on the concentration range studied, and the extrapolated sedimentation coefficient of the monomer was too high, while that of the dimer was too low (thin blue line, **Figure 5A**). In the absence of hydrodynamic models of the protein under study, we suggest that an unlikely small ratio of dimer *s*-value to monomer *s*-value may be a diagnostic tell-tale of a broad transition resulting from heterogeneous preparations of labeled protein. Thus, the ability to study protein concentrations varying more than four orders of magnitude, side-by-side in a single experiment, is a very important advantage of FDS-SV as it can allow us to fully characterize the isotherm. Samples at low concentrations are particularly useful to permit full dissociation of high-affinity subpopulations (or to establish their absence). To this end, in work to be reported in a separate communication, we have explored the lower limits for useful FDS-SV data, and developed techniques to work at low pM concentrations. In the course of that work we have discovered that signal contributions from BSA used as carrier protein, on a level that will not produce visually discernible boundaries, and with amplitudes below the statistical noise of data acquisition, can artificially increase the *s*_w_–values of the dilution isotherm of the protein of interest at very low concentrations, potentially exacerbating a low apparent dimer-to-monomer ratio of the isotherm analysis (Zhao and Schuck unpublished). The data in the present work were corrected for this effect. By contrast, although at high concentrations inner filter effects potentially limit the usefulness of FDS-SV, after appropriate instrument calibration [28] the isotherm can be extended with data acquired using conventional optics to estimate the dimer *s*-value, or titration experiments can be performed using unlabeled protein.

It is remarkable that although both dyes use the same labeling chemistry, and for both conjugation reactions we used similar protein concentrations high above the *K_D_* to protect as much as possible the dimer interface from modification, the use of FAM creates GluA2 ATD subpopulations of higher affinity, unlike Dylight488 labeling or the fusion with EGFP. We believe that data measured using the latter two labels reflects the native dimerization properties of GluA2 ATD because the *K_D_* values of 20.5 and 25.4 nM for Dylight488 and EGFP fusion, respectively, are more consistent with the value of 10 nM reported previously from SV with conventional far-UV detection of unlabeled protein [7], as well as the results from fluorescence anisotropy experiments with Dylight405 [7], and the monomeric state of 1 nM oxazine-labeled GluA2 reported by Jensen et al. [29], all of which indicate that the *K_D_* value of 2 nM obtained for FAM labeled protein is artificially low [16]. The major structural difference between FAM and Dylight488 is the addition of multiple negatively charged sulfonate groups to the fluorescein nucleus in Dylight488; these charged groups reduce hydrophobicity and likely prevent non-specific binding compared to FAM. Perhaps related to this we observed that FAM bound tightly to the resin used for size-exclusion chromatography, requiring extensive washing, while DL-488 eluted within the total column volume. Although it is unclear which residue(s) in the GluA2 ATD when derivatized with FAM adds artificial stability to the dimer, the creation of a hydrophobic interaction surface with the opposing GluA2 ATD protomer would be consistent with the noticeably slower dissociation kinetics of FAM-GluA2 ATD observed directly in the FRET kinetic experiments and inferred from the *c*(*s*) peak pattern. The similarity of the FAM-GluA2 ATD isotherms for different labeling ratios from 0.7 – 2.3 suggests that the increased likelihood of modification of the amino terminal amine might create a higher affinity interaction between GluA2 ATD protomers, that at higher labeling ratios is counterbalanced by additional modifications at other residues responsible for lower affinity dimers. However, we have not studied the structural and energetic details of the FAM-GluA2 ATD further.

In previous work we set out to clarify the origin of the 2,400 range of *K_D_*-values for GluA2 ATD reported in the literature [7]. We thought this to be important not only from the perspective of determining the true affinity of GluA2 ATD dimerization, but also to understand methodological pitfalls of different approaches. The data collected previously on FAM-GluA2 ATD by FDS-SV in the presence of BSA carrier protein, which were consistent with the data reported previously by Rossmann et al. [16], showed features very similar to those presented in the current work, but covered only a 340-fold range of concentration, while in the present study we analyzed a much larger concentration range, 5900 for **Figure 3E/F** and 60,000 for **Figure 4 E/F** and **Figure 5**. We previously noted that the affinity estimates from our prior FDS-SV experiments were questionable because (1) best-fit estimates of monomer sedimentation coefficients were higher than expected, and (2) dimer-to-monomer ratios of sedimentation coefficients were inconsistent with hydrodynamic predictions [7]. These observations remained invariant even after accounting for scan time errors, which we subsequently discovered in the manufacturer’s data acquisition software of the conventional, but not FDS detection systems [30], [31], and therefore confounded the initial comparison of the sedimentation coefficients from the different detection systems. By clarifying the capabilities of the FDS with EGFP as a non-interacting model system, and developing methods to account for its characteristic optical properties [14], we have subsequently also ruled out errors from potential imperfections of the detection system contributing to the observations on FAM-GluA2 ATD, and established that FDS-SV is capable of high accuracy, comparable to or exceeding that of conventional detection systems used in AUC.

The current observation of FAM-induced higher-affinity subpopulations with enhanced dimer life-times can fully explain and resolve these discrepancies: The polydispersity of FAM-GluA2 ATD causes an overly broad overall dimerization isotherm, which is truncated even at the previously rather large concentration range of ∼2.5 decades used in our prior experiments [7]. Therefore, over this concentration range a standard single-component isotherm led to an acceptable fit but with an elevated monomer *s*-value (thin blue line in **Figure 5A**). By adding data points at approximately an order of magnitude lower and higher concentrations (**Figure 5**), and by applying small corrections for the previously unknown fluorescence contributions of the BSA carrier protein, we now find best-fit monomer sedimentation coefficients only slightly higher than those seen for Dylight488-GluA2 ATD. At the same time, the Dylight488-GluA2 ATD isotherm, in contrast to the extended FAM-GluA2 ATD isotherm, fits very well to a model of a homogeneous component with a single affinity constant, and shows that the monomer *s*-value is higher than previously expected based on hydrodynamic modeling, suggesting a more compact conformation than the extended structure for the glycan chains and disordered residues at the amino and carboxy termini previously assumed in the hydrodynamic modeling [7]. Irrespective of the absolute value of the monomer sedimentation coefficient, the estimate of the dimer-to-monomer ratio of *s*-values from the analysis of the Dylight488-GluA2 ATD is significantly higher (s_2_/s_1_ = 1.48) than the value previously obtained [7] from analysis of the FAM-GluA2 ATD isotherm data with a homogeneous dimerization model over a more limited concentration range than used in the present study (s_2_/s_1_ = 1.33). This reinforces that the hydrodynamic parameter estimates from the isotherms can be used to flag problematic experiments and analyses, provided that they are accurately determined.

In an initial attempt to reconcile the previous hydrodynamic parameters from FDS-SV we previously noted a nonlinearity in the signal with loading concentration of FAM-GluA2 ATD, but excluded the possibility that it could quantitatively account for the apparent discrepancies in *s*-values [7], based on theoretical considerations of sedimentation analysis of non-linear signals [14]. We believe it is very useful to critically examine the ratio of (gain-corrected) signal and loading concentration over a wide range of loading concentrations as shown in **Figure 2**, in order to test for signal non-linearity, and to exclude other possible sources of errors in *K_D_* such as oligomeric state-dependent quantum yields. In the present work we did not observe any signal non-linearity, but during the course of our experiments occasionally observed that the high sensitivity of FDS-SV revealed contamination with fluorescent proteins run in previous experiments that artificially alter the signal to concentration ratio, especially at low protein concentrations; such data was discarded. As a result we now pay meticulous attention to cell cleaning when performing FDS-SV experiments. It is also possible that due to the low level of aggregates observed in the current preparations of FAM-GluA2 ATD, and the typical poor reproducibility of aggregation processes in AUC cells, the non-linear response observed previously may have been caused by the formation of rapidly sedimenting aggregates. In any event, from the data in the present work, as well as recent comprehensive follow-up studies on the properties of the FDS system [14], [32], the detection system can be largely ruled out as the source of nonlinearity.

In summary, the present work can explain previous discrepancies of FDS-SV derived affinity constants with those obtained by other methods, as well as the unlikely estimated sedimentation coefficients of monomer and dimer, as artifacts arising from the use of FAM as a fluorescent label. We have shown that a less hydrophobic label, and, similarly, an EGFP fusion protein, provides dimerization constants of GluA2 ATD consistent with data from unlabeled protein. Dylight488, and likely other sulfonated fluorescein derivatives, are not only less hydrophobic, but have the additional advantage of greater photostability, and thus are preferable labels for future studies using FDS-SV. Further experimental design considerations arising from the present work are the need to span a concentration range substantially greater than the usual two orders of magnitude, in order to detect polydispersity in the populations of labeled proteins. FDS-SV is especially useful here because it provides the opportunity for more reliable best-fit end-points of the isotherm as a quality control. In conjunction with improvements in the modeling of raw FDS-SV data accounting for its specific signal characteristics [14], we believe this will aid the maturation of FDS-SV as a reliable quantitative tool for protein interactions. Towards this goal, in a forthcoming communication we will report on a study of the detection limits and a model application to an antibody-antigen interaction with sub-nM *K*_D_ as an example for a high-affinity heterogeneous interaction (Zhao et al, manuscript in preparation).

## Supporting Information

Figure S1 c(s) distributions and *s_w_* isotherms of the titration series of FDS-SV data for the three labels. Panels and symbols are analogous to the dilution isotherm data in Figure 3, with *c*(*s*) analyses and titration isotherm data from Dylight488-GluA2 ATD in Panels A and B, EGFP-GluA2 ATD in Panels C and D, and those from FAM-GluA2 ATD in Panels E and F, respectively.
(TIF)

Figure S2 c(s) distributions of FAM-GluA2 ATD in the presence of different concentrations of BSA. For the BSA concentration dependent assay of FAM-GluA2 ATD, the labeled protein with a labeling ratio of 2.26 was used. Two concentrations of FAM-GluA2 ATD (1 nM in Panel A; 100 nM in Panel B) with a range of BSA concentration (0.1, 0.2, 0.5, 1.0 mg/mL) were examined.
(TIF)

Table S1 Signal weighted-average sedimentation coefficient of FAM-GluA2 ATD in the presence of different concentrations of BSA.
(DOCX)
