# Supplementary figures and images for: Analysis of High Affinity Self-Association by Fluorescence Optical Sedimentation Velocity Analytical Ultracentrifugation of Labeled Proteins: Opportunities and Limitations
Source: PLoS One. 2013 Dec 17;8(12):e83439. doi: 10.1371/journal.pone.0083439 (PMC3866193; doi:10.1371/journal.pone.0083439)

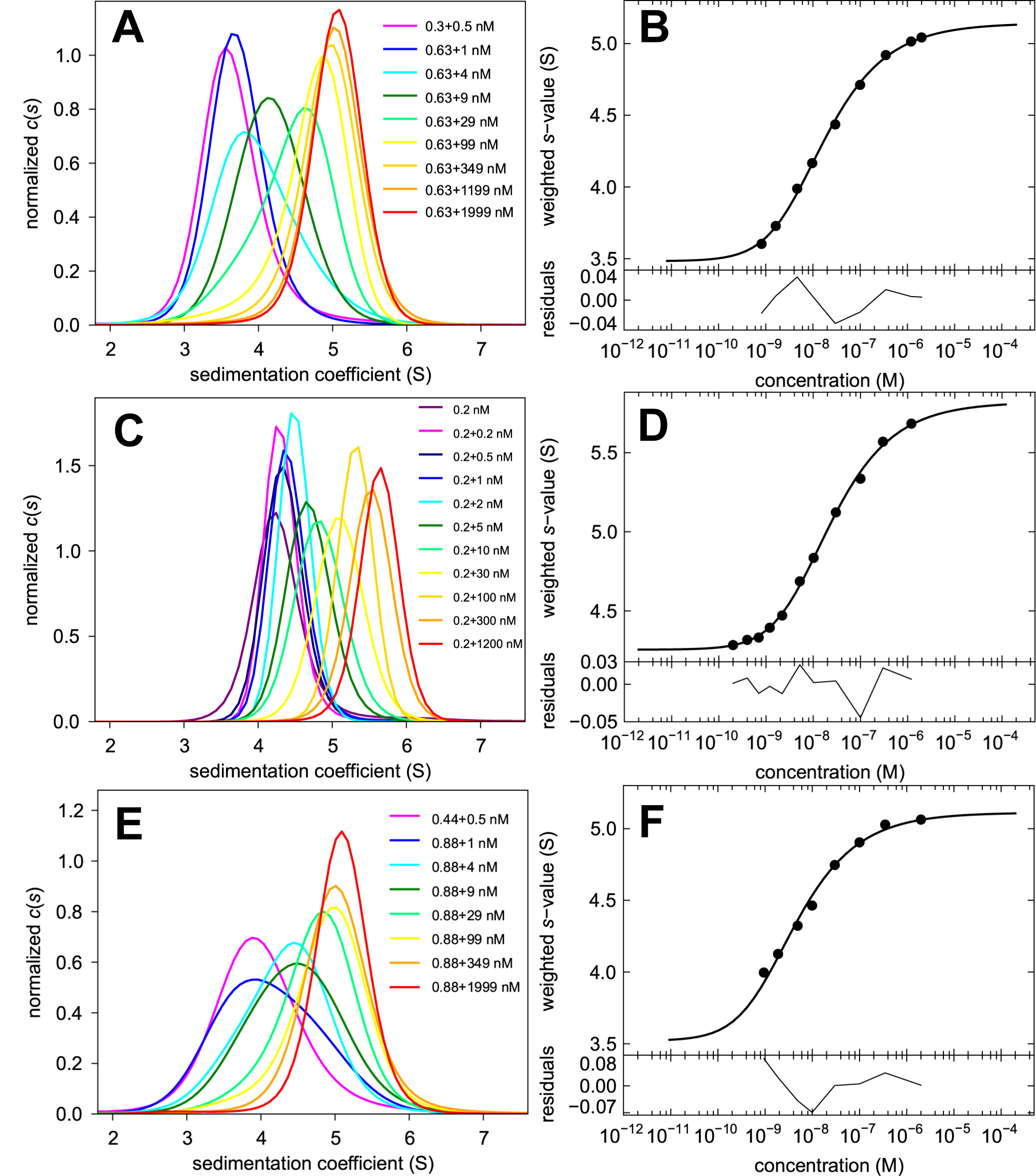

Supplement: Figure S1 — c(s) distributions and sw isotherms of the titration series of FDS-SV data for the three labels. Panels and symbols are analogous to the dilution isotherm data in Figure 3, with c(s) analyses and titration isotherm data from Dylight488-GluA2 ATD in Panels A and B, EGFP-GluA2 ATD in Panels C and D, and those from FAM-GluA2 ATD in Panels E and F, respectively. (TIF) [file pone.0083439.s001.tif]

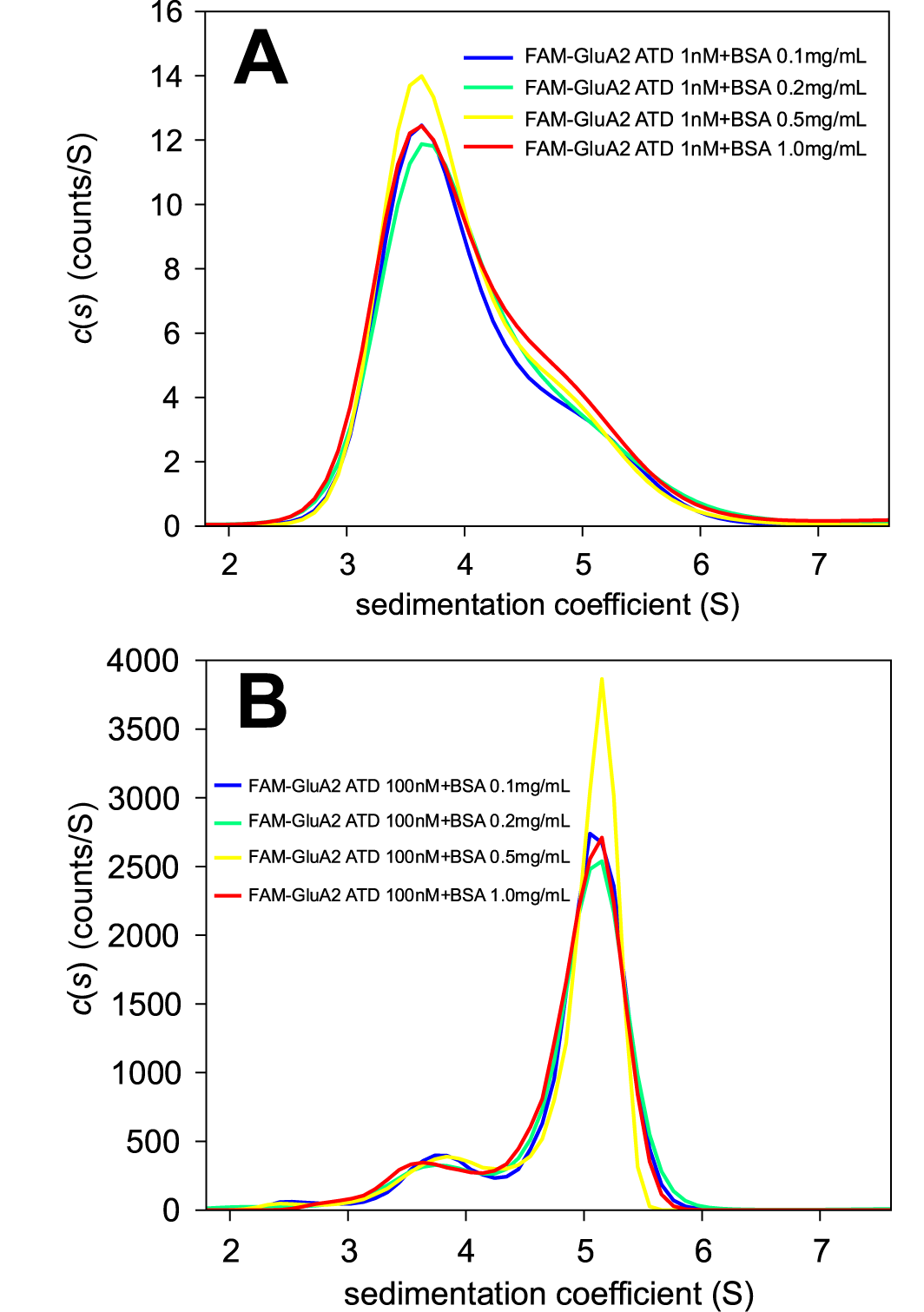

Supplement: Figure S2 — c(s) distributions of FAM-GluA2 ATD in the presence of different concentrations of BSA. For the BSA concentration dependent assay of FAM-GluA2 ATD, the labeled protein with a labeling ratio of 2.26 was used. Two concentrations of FAM-GluA2 ATD (1 nM in Panel A; 100 nM in Panel B) with a range of BSA concentration (0.1, 0.2, 0.5, 1.0 mg/mL) were examined. (TIF) [file pone.0083439.s002.tif]
