# Supplementary material for: Analysis of High Affinity Self-Association by Fluorescence Optical Sedimentation Velocity Analytical Ultracentrifugation of Labeled Proteins: Opportunities and Limitations
Source: PLoS One. 2013 Dec 17;8(12):e83439. doi: 10.1371/journal.pone.0083439 (PMC3866193; doi:10.1371/journal.pone.0083439)
Supplement: Table S1 — Signal weighted-average sedimentation coefficient of FAM-GluA2 ATD in the presence of different concentrations of BSA. (DOCX) [file pone.0083439.s003.docx]

**Supporting Information Table S1**

| [FAM-GluA2 ATD] (nM) | [BSA] (mg/mL) | signal(counts) | s_w_ (S) | rmsd | rmsd/signal | power | Gain |
| --- | --- | --- | --- | --- | --- | --- | --- |
| 0 | 1.0 | 1.359 | 4.860 | 1.391 | 102.4% | 38% | 8 |
| 1 | 0.1 | 16.864 | 4.030 | 1.515 | 9.0% | 38% | 8 |
| 1 | 0.2 | 17.625 | 4.074 | 1.545 | 8.8% | 38% | 8 |
| 1 | 0.5 | 18.448 | 4.022 | 1.532 | 8.3% | 38% | 8 |
| 1 | 1.0 | 18.952 | 4.062 | 1.535 | 8.1% | 38% | 8 |
| 100 | 0.1 | 2106.990 | 4.835 | 7.693 | 0.4% | 38% | 8 |
| 100 | 0.2 | 2116.637 | 4.844 | 7.293 | 0.3% | 38% | 8 |
| 100 | 0.5 | 2117.452 | 4.813 | 8.309 | 0.4% | 38% | 8 |
| 100 | 1.0 | 2132.934 | 4.808 | 8.752 | 0.4% | 38% | 8 |
